# Hybridization and population structure of the *Culex pipiens* complex in the islands of Macaronesia

**DOI:** 10.1002/ece3.307

**Published:** 2012-07-06

**Authors:** Bruno Gomes, Joana Alves, Carla A Sousa, Marta Santa-Ana, Inês Vieira, Teresa L Silva, António PG Almeida, Martin J Donnelly, João Pinto

**Affiliations:** 1 Unidade de Parasitologia Médica, Instituto de Higiene e Medicina Tropical, Universidade Nova de LisboaRua da Junqueira 100, 1349-008, Lisbon, Portugal; 2 Centro de Malária e outras Doenças Tropicais, Instituto de Higiene e Medicina Tropical, Universidade Nova de LisboaRua da Junqueira 100, 1349-008, Lisbon, Portugal; 3 Direcção-Geral da Saúde Ministério da SaúdePalácio do Governo, CP 47, Praia, Cabo Verde; 4 Unidade de Parasitologia e Microbiologia Médicas, Instituto de Higiene e Medicina Tropical, Universidade Nova de LisboaRua da Junqueira 100, 1349-008, Lisbon, Portugal; 5 Centro de Estudos da Macaronésia, Universidade da MadeiraCampus da Penteada, 9000-390, Funchal, Portugal; 6 Vector Group, Liverpool School of Tropical MedicinePembroke Place, Liverpool, L3 5QA, UK

**Keywords:** *Culex pipiens*, *Culex quinquefasciatus*, hybridization, Macaronesian islands, West Nile virus

## Abstract

The *Culex pipiens* complex includes two widespread mosquito vector species, *Cx. pipiens* and *Cx. quinquefasciatus*. The distribution of these species varies in latitude, with the former being present in temperate regions and the latter in tropical and subtropical regions. However, their distribution range overlaps in certain areas and interspecific hybridization has been documented. Genetic introgression between these species may have epidemiological repercussions for West Nile virus (WNV) transmission. Bayesian clustering analysis based on multilocus genotypes of 12 microsatellites was used to determine levels of hybridization between these two species in Macaronesian islands, the only contact zone described in West Africa. The distribution of the two species reflects both the islands' biogeography and historical aspects of human colonization. Madeira Island displayed a homogenous population of *Cx. pipiens*, whereas Cape Verde showed a more intriguing scenario with extensive hybridization. In the islands of Brava and Santiago, only *Cx. quinquefasciatus* was found, while in Fogo and Maio high hybrid rates (∼40%) between the two species were detected. Within the admixed populations, second-generation hybrids (∼50%) were identified suggesting a lack of isolation mechanisms. The observed levels of hybridization may locally potentiate the transmission to humans of zoonotic arboviruses such as WNV.

## Introduction

The biological diversity of islands with recent volcanic origin and high isolation from mainland is a result of the colonizers ability to break the isolation and survive the island's environmental conditions. The highly stochastic nature of colonizing events means that only a very limited number of taxa may be present in each archipelago (Gillespie and Roderick 2002). For example, in Hawaii, only 15% of the known insect families were observed (Howarth and Ramsay 1991), and a similar scenario occurs in the Macaronesian region (Juan et al. 2000; Gillespie and Roderick 2002). This region is formed by four archipelagos of volcanic islands located in the northern hemisphere of the Atlantic Ocean: Azores, Canary Islands, Cape Verde, and Madeira. Isolation and low colonization rates in these islands promote divergence by adaptive radiation, leading to a higher proportion of neoendemic species than in regions with lower levels of genetic isolation (Gillespie and Roderick 2002). In Macaronesia, there are several examples of adaptive radiations in vertebrate species such as lizards (Gallotiinae, Gekkonidae, and Scincidae; Carranza et al. 2001, 2002; Carranza and Arnold 2006; Cox et al. 2010) and invertebrates such as beetles (*Calathus*, *Meladema, Pimelia, Tarphius*), butterflies (*Gonepteryx*), and spiders (*Pholcus*; Brunton and Hurst 1998; Emerson et al. 2000a,b; Contreras-Diaz et al. 2003; Ribera et al. 2003; Dimitrov et al. 2008). However, rates of island endemism appear to be lower for mosquitoes (Diptera: Culicidae). Of the 11 mosquito species/subspecies found in the Canary Islands, only two are endemic for Macaronesia and these are shared with Madeira (Capela 1982; Báez and Oromí 2010). This contrasts with the nearly 50% endemism rate among terrestrial invertebrate species in Canary Islands (Juan et al. 2000). A similarly low proportion of endemic mosquitoes is observed in other volcanic islands such as Cape Verde and Hawaii (Shroyer 1981; Alves et al. 2010). The reason for the relative paucity of adaptive radiation in island mosquito populations is that they are very recent colonizers often as a result of multiple human-mediated introductions (Fonseca et al. 2000; Lounibos 2002; Bataille et al. 2009).

Invasions of certain mosquito species can have a negative impact in vertebrates and humans due to their ability to serve as transmission vectors of diseases (Lounibos 2002; Delatte et al. 2011). A remarkable example was the decline of native bird' populations in Hawaii associated with avian malaria and avian pox virus transmitted by the introduced mosquito vector *Culex quinquefasciatus* Say, 1823 (Fonseca et al. 2000; Lapointe et al. 2012).

**Figure 1:**
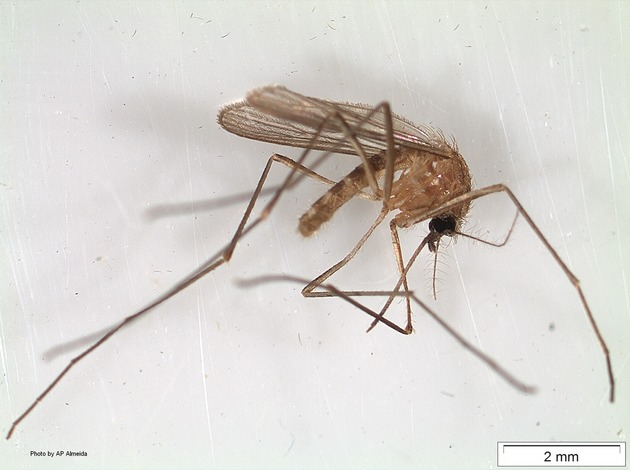
Mosquito (female) of the *Culex pipiens* complex. Photograph with a digital camera SC30 (OLYMPUS, Tokyo, Japan) under a stereomicroscope OLYMPUS SZ61 (12× magnification).

The *Culex pipiens* complex (Fig. 1) comprises mosquito vectors responsible for the transmission of lymphatic filariasis and neurotropic arboviruses from the Japanese encephalitis serogroup including the West Nile virus (WNV) to humans (Smith and Fonseca 2004; Solomon 2004). The nominal species of the complex, *Culex pipiens* Linnaeus, 1758 sensu stricto (hereafter termed *Cx. pipiens*) and *Cx. quinquefasciatus* are the most common and widespread species. The former is found primarily in temperate zones, whereas the latter occurs in tropical and subtropical zones. *Cx. pipiens* has a greater ecological range with populations found from the low subarctic of Siberia and Scandinavian countries to the semidesert regions of the Maghreb (Vinogradova 2000). *Cx. quinquefasciatus* is confined to warmer tropical and subtropical regions with a higher degree of humidity (Subra 1981; Fonseca et al. 2006). However, it is possible to find regions where both species coexist sympatrically and where hybrids of the two species have been observed (Urbanelli et al. 1995; Humeres et al. 1998; Kothera et al. 2009; Alves et al. 2010).

In North America, morphological identification of males based on the length of the dorsal and ventral arms of the phallosome, namely the DV/D ratio, revealed the presence of only *Cx. pipiens* at latitudes above 39ºN while *Cx. quinquefasciatus* was the only species found at latitudes below 36ºN (Barr 1957). In the areas between 36ºN and 39ºN, a hybrid zone between the two species has been described (Barr 1957; Savage et al. 2008). Females are morphologically indistinguishable, and several molecular methods have been described to identify these sibling species (Farajollahi et al. 2011). Of these, the PCR assay based on species-specific polymorphisms in the intron-2 of the acetylcholinesterase-2 gene (ACE-2) has been one of the most widely used (Smith and Fonseca 2004). Allozyme studies confirmed the latitudinal cline between the two species (Cornel et al. 2003). A recent microsatellite-based study extended the geographic limits of “Barr's hybrid zone” suggesting a wider area between 30ºN and 40ºN (Kothera et al. 2009).

In contrast with the American continent, isolation between *Cx. pipiens* from Mediterranean Europe and *Cx. quinquefasciatus* from the northern hemisphere of Africa appears to be absolute. The most plausible explanation for this isolation is the presence of the Sahara desert. This inhospitable region lying between 15ºN and 33ºN acts as a barrier to gene flow not only for insects but also for other organisms (Douady et al. 2003; Kodandaramaiah and Wahlberg 2007). An exception is likely to be found in the Macaronesian region. Madeira, the Canary Islands, and Cape Verde locate within the latitudinal interval of the Saharan desert. In spite of the influence of the Saharan winds, the islands that compose these archipelagos have quite varying climates, ranging from temperate with dry summers (Madeira: Csb, Köppen Classification System) to arid with hot temperatures (Cape Verde: BWh; Peel et al. 2007). Importantly, many of these islands display environmental conditions for sustaining mosquito populations.

Populations of *Cx. pipiens* have been identified in the four archipelagos, and *Cx. quinquefasciatus* has been recorded in Cape Verde (Capela 1982; Alves et al. 2010; Báez and Oromí 2010; Vieira et al. 2010). The observation of intermediate DV/D values for the male genitalia of some specimens from Cape Verde suggested the presence of hybrids (Ribeiro et al. 1980). Similarly, in a recent update on the mosquito fauna of Cape Verde, molecular identification based on the ACE*-*2 marker suggested hybrid frequencies of 39–67% in two islands of the archipelago (Alves et al. 2010). However, the extent of hybridization and genetic introgression between *Cx. pipiens* and *Cx. quinquefasciatus* in these islands is still largely unknown.

There are several examples of species expansion mediated by human activity that have broken the geographic isolation between sibling species of insects and other organisms (Pinto et al. 2005; Steeves et al. 2010). The lack of other isolation mechanisms between these species may allow introgression leading to species assimilation or erosion of species boundaries. There is evidence supporting that two isolation mechanisms between *Cx. pipiens* and *Cx. quinquefasciatus* are likely to occur: (1) prezygotic isolation may result from differences in species distribution and in mating behavior; and (2) intrinsic postzygotic may result from cytoplasmic incompatibility that creates unviable hybrids (Vinogradova 2000; Cornel et al. 2003). A role of extrinsic postzygotic mechanisms linked to hybrid fitness (McBride and Singer 2010) in the isolation of the *Cx. pipiens* complex members remains unclear.

Both species also display important bio-ecological differences. *Culex quinquefasciatus* is generally considered a more synanthropic urban mosquito compared with a more rural *Cx. pipiens* (Ribeiro et al. 1980; Subra 1981). In Brazil and in Africa, *Cx. quinquefasciatus* displays a strong preference for mammals (including humans; Subra 1981; Muturi et al. 2008; Lorosa et al. 2010). In North America, there are differences in host preference among populations of *Cx. quinquefasciatus*, with some preferring mammals (Zinser et al. 2004; Molaei et al. 2007) and others birds (Savage et al. 2007; Molaei et al. 2010). *Culex pipiens* preferentially feeds upon birds (Kilpatrick et al. 2006, 2007; Molaei et al. 2006). Hybridization between members of the *Cx. pipiens* complex with different host preferences may promote a more opportunistic feeding behavior increasing the importance of the host availability (Fonseca et al. 2004; Balenghien et al. 2011). Consequently, this population with more catholic feeding behavior would have an increased potential as a bridge vector between bird and humans for the transmission of WNV (Molaei et al. 2007; Savage et al. 2007; Kilpatrick 2011).

In this study, Bayesian model-based methods were applied to multilocus microsatellite genotypes to infer the genetic structure of the *Cx. pipiens* complex in Madeira and in four islands of Cape Verde. The aims were (1) to determine the degree of genetic differentiation among island populations; (2) to measure rates of hybridization and genetic introgression between the sibling species; and (3) to infer about the colonization process and their impacts in Macaronesian region.

## Materials and Methods

### Mosquito collections

**Figure 2:**
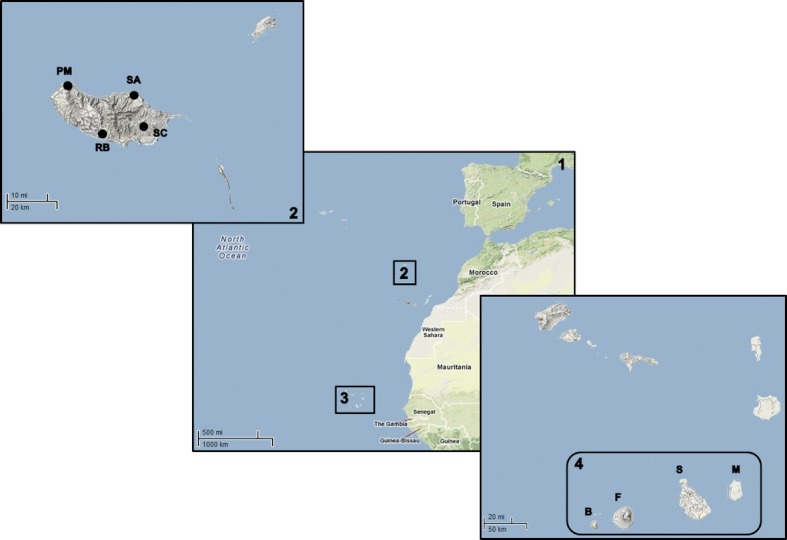
Maps of the North Atlantic region showing the localities/islands sampled. (1) North Atlantic region including West Africa and Mediterranean region; (2) Madeira archipelago, PM, Porto Moniz; RB, Ribeira Brava; SC, Santa Cruz; SA, Santana; (3) Cape Verde archipelago; (4) Islands sampled in Cape Verde; B, Brava; F, Fogo; S, Santiago; M, Maio. Images collected in Google Maps – ©2012 Google (http://maps.google.com/).

Indoor resting collections of adult mosquitoes using mechanical aspirators were carried out in four localities of Madeira Island in September 2006 and in June 2007 (Fig. 2). Given the very low adult mosquito densities found in Cape Verde (Pinto et al. 1999), collections of immature culicids were undertaken using dippers and pipettes, between November and December 2007, in four islands of Cape Verde: Brava, Fogo, Santiago, and Maio (Fig. 2). Immature collections were made on a wide range of breeding sites such as ponds, pools, swamps, pits, water tanks, and septic tanks. Information on the localities in which *Cx. pipiens* s.l. larvae were sampled in Cape Verde is shown in Table S1 (Supporting information; see also Alves et al. 2010). Collected larvae were transported to a laboratory in Praia (Santiago Island) and reared to adulthood. Adult mosquitoes were killed by freezing and identified to species/complex using morphological keys (Ribeiro and Ramos 1995, 1999). Samples were stored over silica gel until DNA extraction.

### Molecular analyses

DNA extraction from individual female mosquitoes was performed using the method of Collins et al. (1987). Each specimen was identified to species by a multiplex PCR assay targeting species-specific polymorphisms in intron-2 of the ACE-2 gene using primers specific for *Cx. pipiens*, *Cx. quinquefasciatus*, and *Cx. torrentium* (Smith and Fonseca 2004).

Twelve microsatellites (Fonseca et al. 1998; Keyghobadi et al. 2004; Smith et al. 2005) were genotyped (see Table S2). For each specimen, each locus was amplified separately in a 20 μL PCR reaction that contained 1X GoTaq® Flexi Buffer (Promega, Madison, Wisconsin), 2.5 mm MgCl_2_, 0.25 mm dNTPs, 0.20 mg/mL Bovine Serum Albumin, 0.20 μm of each primer, and 0.5 U GoTaq® Flexi DNA polymerase (Promega). For each locus, one of the primers was fluorescently labeled (NED, HEX our 6-FAM; Applied Biosystems, Carlsbad, California). Thermocycling conditions included an initial denaturation step of 5 min at 96ºC, followed by 30 cycles each of 96ºC for 30 sec, annealing at 52ºC-56ºC (locus-dependent) for 30 sec and 72ºC for 30 sec. After a final extension step of 5 min at 72ºC, reactions were stopped at 4ºC.

Amplified products were separated by capillary electrophoresis in a genetic analyzer ABI3730 (Applied Biosystems) at Yale DNA Analysis Facility (USA). Fragment sizes and genotypes were scored using the software GeneMarker 1.4. (Softgenetics, State College, Pennsylvania).

### Data analysis

Genetic diversity at each microsatellite locus was characterized by estimates of expected heterozygosity (Nei 1987) and inbreeding coefficient (*F*_IS_). Significance of *F*_IS_ values was assessed by randomization tests. These analyses were performed using FSTAT v. 2.9.3.2. (Goudet 1995). Estimates of allele richness (*A*_R_), a measure of allele diversity adjusted for the lowest sample size, were obtained by the statistical rarefaction approach implemented in HP-RARE (Kalinowski 2005). Departures from Hardy–Weinberg proportions were tested by exact tests available in ARLEQUIN v.3.11 (Excoffier et al. 2005). The same software was used to perform exact tests of linkage equilibrium between pairs of loci based on the expectation-maximization approach described by Slatkin and Excoffier (1996). The software Micro-Checker 2.2.3. (Van Oosterhout et al. 2004) was used to test for the presence of null alleles (99% confidence interval) at each locus/sample.

Bayesian clustering analysis as implemented by STRUCTURE 2.3.3 (Pritchard et al. 2000) was used to infer population substructure/ancestry from the data set without prior information of sampling groups under the conditions of admixture (α allowed to vary between 0 and 10), and allele frequencies correlated among populations (λ was set at 1, default value). Ten independent runs with 10^5^ iterations and replications were performed for each value of *K* (*K* = 1–10 clusters). The inference of the number of genetic clusters (*K*) in the Bayesian method implemented by STRUCTURE is not straightforward, and it is normally performed by ad hoc approaches: an estimation of ln[Pr(*X*|*K*)], described in the original publication (Pritchard et al. 2000) and the Δ*K* statistic (Evanno et al. 2005). We used a combination of these approaches with a sequential procedure in which data were analyzed at three levels: (1) all samples, (2) each archipelago, and (3) each island. Following the suggestions of Vähä and Primmer (2006), individual genetic assignment to clusters was based on a minimum posterior probability threshold (*Tq*) of 0.90. Individuals displaying 0.1 ≤ *q*_*i*_ ≤ 0.90 were considered of admixed ancestry. The information from the outputs of each *K* (10 runs) was aligned by the Greedy method implemented in CLUMPP (Jakobsson and Rosenberg 2007).

The Bayesian method implemented by NEWHYBRIDS 1.1. (Anderson and Thompson 2002) was used to assign individuals into six classes: two pure (parental *Cx. pipiens* and *Cx. quinquefasciatus*) and four hybrid (F1, F2, and backcrosses with the parental populations). The approach of uniform priors was used because it reduces the influence of low-frequency alleles thus which may result from sampling and genotyping errors in closely related populations. Results were based on the average of five independent runs of 10^5^ iterations. Following the suggestions of Anderson and Thompson (2002), individual genetic assignment to classes was based on a minimum posterior probability threshold (*Tq*) of 0.50.

A neighbor-joining (NJ) tree based on Cavalli-Sforza and Edwards (1967) chord distance (Dc) was used to represent the relationships among genetic clusters and geographic samples. Individuals with an admixed genetic background (i.e. with a probability of assignment not attributable to any of the purebred or hybrid clusters) were excluded from this analysis. A consensus tree was obtained by bootstrapping (1000 replicates) distance values over loci. Calculations were performed with the program Populations 1.2.30 (Langella 1999). The software Treeview (Page 1996) was used to visualize the tree.

Whenever multiple testing was performed, the nominal significance level of rejection of the null hypothesis (α = 0.05) was corrected by the sequential Bonferroni procedure (Holm 1979).

## Results

### ACE-2 molecular identification

A total of 374 females (Madeira: 190 and Cape Verde: 184, distributed as follows, Brava: 31, Fogo, 36, Santiago: 54, Maio: 63) were analyzed by the molecular assay ACE-2 (Smith and Fonseca 2004; Table 1). Of these, 203 were identified as *Cx. pipiens* and were collected in Madeira (*N* = 190) and in Maio (*N* = 13). *Culex quinquefasciatus* was found in the four islands of Cape Verde (*N* = 115), and it was the only member of the complex present in the collections from Brava (*N* = 31) and Santiago (*N* = 54). Fifty-six mosquitoes displayed a heterozygous pattern for ACE-2 and were collected in Fogo (*N* = 14) and Maio (*N* = 42). The island of Maio was the only island where the two species and putative hybrids were found in sympatry.

**Table 1:** Molecular identification of *Culex pipiens* complex species based on the molecular assay in the ACE-2

		Localities							
		
		Madeira				Cape Verde			
			
	*N*	PM	RB	SC	SA	B	F	S	M
*Culex pipiens*	203	66 (100.0)	39 (100.0)	34 (100.0)	51 (100.0)	0 (0.0)	0 (0.0)	0 (0.0)	13 (20.6)
Hybrids	56	0 (0.0)	0 (0.0)	0 (0.0)	0 (0.0)	0 (0.0)	14 (38.9)	0 (0.0)	42 (66.7)
*Culex quinquefasciatus*	115	0 (0.0)	0 (0.0)	0 (0.0)	0 (0.0)	31 (100.0)	22 (61.1)	54 (100.0)	8 (12.7)

*N*, number of individuals; PM, Porto Moniz; RB, Ribeira Brava; SC, Santa Cruz; SA, Santana; B, Brava island; F, Fogo island; S, Santiago island; M, Maio island.

Values in parenthesis refer to the relative genotypic frequencies (in percentage) within each locality.

### Clustering analysis

Genetic diversity estimates for the 12 microsatellites in whole sample (*N* = 374) and subsamples determined by ACE-2 identification and geographic location are shown in Table S3. Loci CQ26 and CQ41 exhibited heterozygote deficits in all subsamples from Madeira, possibly reflecting locus-specific effects, such as null alleles or selection. The analysis performed by the Micro-Checker software confirmed the possibility of null alleles at loci CQ26 and CQ41 in samples from Madeira island (see Table S3). These loci were therefore excluded from Bayesian assignment and genetic differentiation analyses.

**Figure 3:**
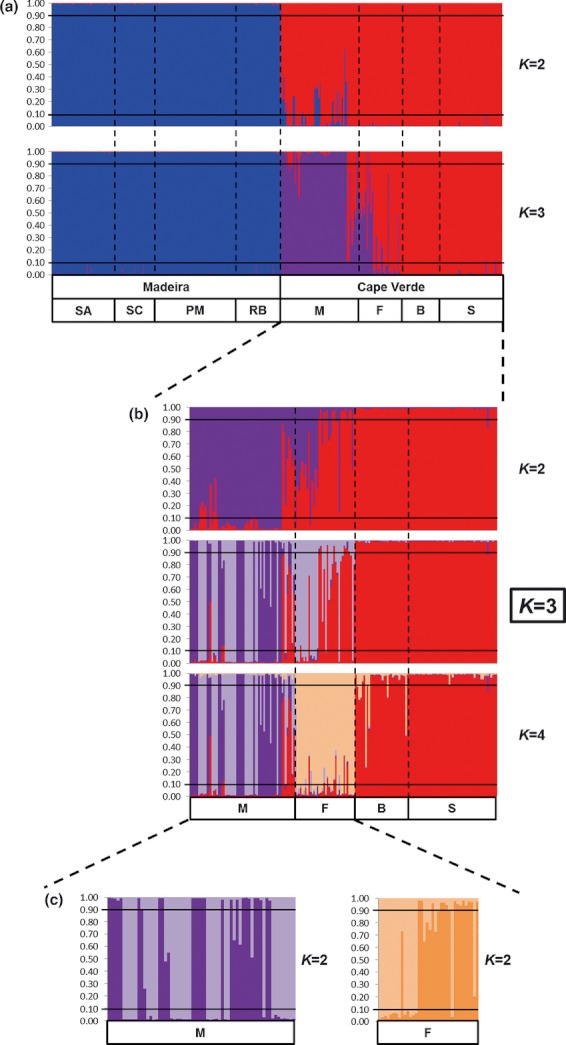
Bayesian cluster analysis conducted by STRUCTURE at three different levels. (a) all samples, (b) Cape Verde samples, (c) Maio and Fogo islands. *K*, number of clusters. Columns correspond to the multilocus genotype of each individual, partitioned in different colors representing the probability of ancestry (qi) to each cluster. Individuals were ordered according to their geographic information. Lines indicate the qi threshold used to determine admixed individuals (see Methods).

The Bayesian analysis implemented in STRUCTURE and the two ad hoc approaches to define the number of clusters revealed a homogeneous population in Madeira (*K* = 1) and the intriguing scenario of Cape Verde with three possible subdivisions (*K* = 2, *K* = 3, or *K* = 4; Fig. 3, see Figs. S1, S2). The sequential procedure under the three levels of organization (whole sample, archipelago, and island) highlighted a further subdivision within the islands of Maio and Fogo providing support for *K =* 3 in the archipelago of Cape Verde and consequently a *K* = 4 for the whole sample (Fig. 3, see Fig. S3).

The combination of the Bayesian clustering results with the ACE-2 identification clarified the separation of sampled mosquitoes into four different clusters (Table 2): Cluster 1 (C1) grouped all the 190 *Cx. pipiens* from Madeira, while the other three clusters were restricted to Cape Verde. Cluster 4 (C4) was the most abundant in the archipelago with 91 specimens from three islands (Brava, Fogo and Santiago), all identified as *Cx. quinquefasciatus* by ACE-2 PCR. Cluster 2 (C2) was the smallest cluster with 25 specimens from Maio Island being classified as *Cx. pipiens* or hybrid by ACE-2 PCR. Cluster 3 (C3) includes individuals from Fogo and Maio Islands, and the majority (87.2%) of the specimens were identified as hybrids by ACE-2 PCR. Twenty-nine specimens, the majority of which from Maio and Fogo, were not assigned to any of the four clusters and were thus considered admixed.

**Table 2:** ACE-2 PCR species composition and relative distribution per locality/island of each genetic cluster revealed by STRUCTURE

		ACE-2			Localities							
			
					Madeira				Cape Verde			
						
	*N*	P	H	Q	PM	RB	SC	SA	B	F	S	M
Cluster 1	190	190 (100.0)	0 (0.0)	0 (0.0)	66 (34.7)	39 (20.5)	34 (17.8)	51 (26.8)	0 (0.0)	0 (0.0)	0 (0.0)	0 (0.0)
Cluster 2	25	9 (36.0)	16 (64.0)	0 (0.0)	0 (0.0)	0 (0.0)	0 (0.0)	0 (0.0)	0 (0.0)	0 (0.0)	0 (0.0)	25 (100.0)
Cluster 3	39	1 (2.6)	34 (87.2)	4 (10.2)	0 (0.0)	0 (0.0)	0 (0.0)	0 (0.0)	0 (0.0)	14 (35.9)	0 (0.0)	25 (64.1)
Cluster 4	91	0 (0.0)	0 (0.0)	91 (100.0)	0 (0.0)	0 (0.0)	0 (0.0)	0 (0.0)	31 (34.1)	7 (7.7)	53 (58.2)	0 (0.0)
Admixed	29	3 (10.3)	6 (20.7)	20 (69.0)	0 (0.0)	0 (0.0)	0 (0.0)	0 (0.0)	0 (0.0)	15 (51.6)	1 (3.4)	13 (44.8)

*N*, number of individuals; P, *Culex pipiens* by ACE-2 identification; Q, *Culex quinquefasciatus* by ACE-2 identification; H, hybrids between *Culex pipiens* and *Culex quinquefasciatus* by ACE-2 identification; PM, Porto Moniz; RB, Ribeira Brava; SC, Santa Cruz; SA, Santana; B, Brava island; F, Fogo island; S, Santiago island; M, Maio island.

Values in parenthesis refer to the frequencies (in percentage) within each cluster.

The analysis with NEWHYBRIDS confirmed the homogeneity of the Madeira population (C1). In Cape Verde, all the samples from C4 were classified as pure *Cx. quinquefasciatus*, while the majority of the individuals of C2 (96.0%) were classified as pure *Cx. pipiens* and one individual was classified as a backcross with *Cx. pipiens* (BxP). The majority of individuals of C3 (87.1%) were classified as hybrids. Of these, 10 (nine in Fogo, one in Maio) were classified as F2 hybrids and nine individuals from Maio were backcrosses with *Cx. pipiens* (BxP; Table 3).

**Table 3:** Frequencies purebred and hybrid individuals detected by NEWHYBRIDS in each of the ancestry clusters revealed by STRUCTURE

		NEWHYBRIDS							
		
					H				
					
	*N*	P	Q	H	F1	F2	BxP	BxQ	H′
Cluster 1	190	189 (99.4)	0 (0.0)	1 (0.6)	0 (0.0)	0 (0.0)	0 (0.0)	0 (0.0)	1 (0.6)
Cluster 2	25	24 (96.0)	0 (0.0)	1 (4.0)	0 (0.0)	0 (0.0)	1 (4.0)	0 (0.0)	0 (0.0)
Cluster 3	39	4 (10.3)	1 (2.6)	34 (87.1)	0 (0.0)	10 (25.6)	9 (23.1)	0 (0.0)	15 (38.4)
Cluster 4	91	0 (0.0)	91 (100.0)	0 (0.0)	0 (0.0)	0 (0.0)	0 (0.0)	0 (0.0)	0 (0.0)
Admixed	29	3 (10.4)	13 (44.8)	13 (44.8)	0 (0.0)	8 (27.6)	0 (0.0)	0 (0.0)	5 (17.2)

*N*, number of individuals; P, pure *Culex pipiens*; Q, pure *Culex quinquefasciatus*; H, hybrids between the pure groups (*Culex pipiens* and *Culex quinquefasciatus*); F1, hybrid first generation; F2, hybrids second generation; BxP, backcross *Culex pipiens*; BxQ, backcross *Culex quinquefasciatus*, H′, hybrids defined by the sum of assignment probabilities for all hybrid classes.

Values in parenthesis refer to the frequencies (in percentage) within each cluster.

**Figure 4:**
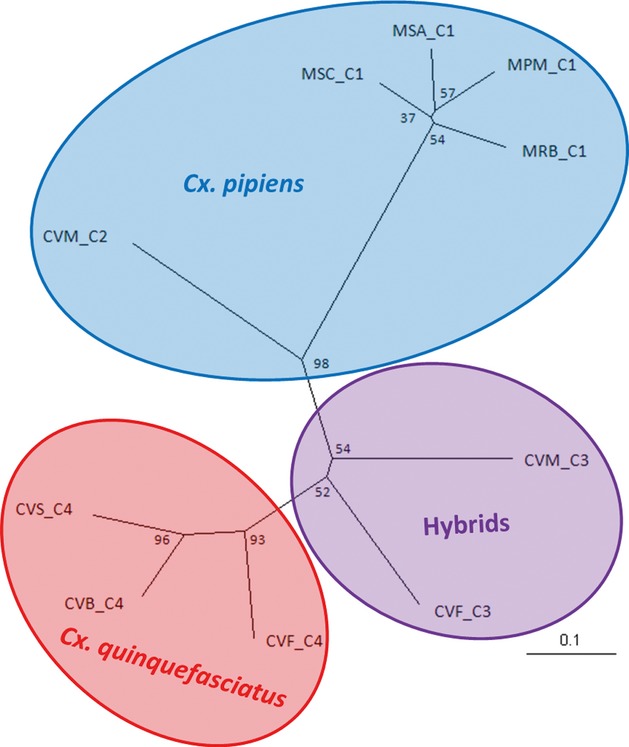
Phylogenetic tree of the *Culex pipiens* complex in Madeira and Cape Verde. CVM, Maio; CVF, Fogo; CVB, Brava; CVS, Santiago; MSC, Santa Cruz (Madeira); MSA, Santana (Madeira); MPM, Porto Moniz (Madeira); MRB, Ribeira Brava (Madeira); C1, cluster 1; C2, cluster 2; C3, cluster 3 and C4, cluster 4.

The Dc-based NJ tree was consistent with the presence of the four clusters identified in the analysis performed by STRUCTURE (Fig. 4). *Culex pipiens* samples from Madeira (C1) and Maio (C2) displayed a high genetic distance but were still grouped in a common cluster separated from the remaining samples. Samples from cluster C3, composed mainly by hybrid individuals, displayed an intermediate position in the topology of the tree. *Culex quinquefasciatus* samples from cluster C4 shared the same cluster, but it was possible to observe significant divergence between the populations of the three islands (Brava, Fogo, and Santiago).

## Discussion

In this study, the distribution and levels of hybridization between *Cx. pipiens* and *Cx. quinquefasciatus* were found to differ among islands of the Macaronesian region. Madeira showed a genetically homogenous *Cx. pipiens* population. In Cape Verde, it was possible to identify monospecific populations of *Cx. quinquefasciatus* in Brava and Santiago, while admixed populations between both species were observed in Maio and Fogo. The species diagnostic ACE-2 PCR was effective in the identification of each species in the allopatric populations of Madeira, Brava, and Santiago. However, in sympatric populations with interspecific admixture such as those of Maio and Fogo, repeated introgression and recombination lead to a disruption of the linkage between the diagnostic alleles and the respective genetic backgrounds of each species. Under these conditions, a more cautious interpretation of the results obtained by a single diagnostic marker such as the ACE-2 is needed for the correct identification of each species and hybrids (McAbee et al. 2008; Fonseca et al. 2009).

The presence of a monospecific population of *Cx. pipiens* in Madeira agrees with previous reports (Capela 1981; Fonseca et al. 2004). This volcanic island locates in the temperate zone of the North Atlantic, 700 km off the coast of Morocco and 850 km from continental Portugal. In both countries, only *Cx. pipiens* has been identified (Trari et al. 2002; Almeida et al. 2008). Since its discovery in the 15th century, Madeira has been an important port-of-call in the Atlantic so that the introduction of *Cx. pipiens* could have resulted from human-mediated passive dispersal (Lounibos 2002). The Mediterranean temperate climate of this island should also be compatible with the establishment of *Cx. pipiens*. The absence of *Cx. quinquefasciatus* may reflect a lower tolerance of this species to more temperate climates with lower temperatures during winter. The possibility of this vector having never been introduced into this island is probably less likely in spite of the *ca*. 2000-km distance between Madeira island and the sub-Saharan African coast. Migration by human-mediated dispersal in *Cx. pipiens* complex includes long-distance introductions that spread organophosphate insecticides resistance genes between continents and established invasive populations in isolated archipelagos such as Hawaii (Chevillon et al. 1999; Fonseca et al. 2006).

The distribution of the two members of the *Cx. pipiens* complex in Cape Verde is more intricate and reflects both bio-geographic features and historical aspects of the human peopling of the islands. The apparent predominance of *Cx. quinquefasciatus* on the archipelago agrees with the bio-geographic context of the islands, which lie in the tropical zone of the North Atlantic. The most likely origin of this species would be the West African continent. However, Fonseca et al. (2006) in a worldwide genetic survey of *Cx. quinquefasciatus* provided evidence for a recent introduction of *Cx. quinquefasciatus* in West Africa from the New World. Given the geographic intermediate location and the strategic importance of Cape Verde in maritime routes, one cannot exclude the possibility of introduction of mosquitoes of New World origin. The occurrence of *Cx. pipiens* most likely reflects the historical relationship of the archipelago with the Mediterranean region of the European continent. The islands were discovered by Portuguese sailors in the 15th century, and the subsequent peopling was made by migrants of both European and African origin. The Portuguese traders used the archipelago as a port-of-call for ship provisioning during travels between Europe and the African continent and also as a Senegambian slave outpost for the Atlantic (Brehm et al. 2002). The considerable movement of ships between the islands and both continents could have provided the opportunity for the introduction of both mosquito species. It remains to be determined whether the present mosquito populations in Cape Verde islands result from multiple introduction events of one or both species. The analysis of mainland samples and of other molecular markers (e.g. mtDNA) would be required for this purpose (Hardouin et al. 2010).

In Maio and Fogo, a considerable number of individuals were assigned as hybrids and yet one of the parental species was absent from the samples (*Cx. quinquefasciatus* in Maio; *Cx. pipiens* in Fogo). A possible explanation for this apparent contradiction could be insufficient sampling of the least abundant species. Factors that may have contributed for an insufficient sampling were the collection method used (immature captures) coupled with the low number of breeding sites positive for *Cx. pipiens s.l*. larvae. In Cape Verde, very low adult mosquito densities preclude the use of collection methods targeting adult mosquitoes for sampling sufficient numbers of individuals (Ribeiro et al. 1980; Pinto et al. 1999). However, other explanations may be proposed for these observations. Maio is the driest of the islands sampled and a lower density or virtual absence of a stable *Cx. quinquefasciatus* population agrees with its lower tolerance to aridity. Fogo has a very steep topography marked by the presence of a volcanic cone. A similar scenario to that of Madagascar, where *Cx. quinquefasciatus* predominated in the lowland urban areas and *Cx. pipiens* were found at altitudes above 1300 m (Urbanelli et al. 1995), may occur in this island. Insufficient sampling could also explain the apparent absence of *Cx. pipiens* in Brava and Santiago, although in these cases there was no evidence of admixture. While the absence of this species agrees with previous surveys in Brava, the same does not hold for Santiago. In this island, *Cx. pipiens* prevailed over *Cx. quinquefasciatus* in larval collections performed in the late 1970s (Ribeiro et al. 1980). This apparent inversion in the relative abundance of both species may be associated with an increase in urbanization of this island over the past recent years. Such an increase in urbanization could confer a greater adaptive advantage for *Cx. quinquefasciatus* over *Cx. pipiens*.

High hybridization rates between *Cx. pipiens* and *Cx. quinquefasciatus* were detected in two islands of Cape Verde. These rates (Fogo: 39%; Maio: 40%) are comparable with those recorded in the hybrid zone of North America (Savage et al. 2008; Kothera et al. 2009) and contrast with the pattern of sympatry without hybridization observed in southeast Africa (Cornel et al. 2003). The lack of hybridization in southeast Africa was justified by the presence of *Wolbachia pipientis* only in *Cx. quinquefasciatus,* whereas in North America, both species are infected with the same strain (Cornel et al. 2003; Rasgon and Scott 2003)*. Wolbachia pipientis* infection can induce sterility by cytoplasmic incompatibility (an intrinsic postzygotic isolation mechanism) between infected males and uninfected females or females infected by incompatible strains (Atyame et al. 2011). In West Africa and in the Mediterranean region, both *Cx. pipiens* and *Cx. quinquefasciatus* populations share closely related strains of *W. pipientis* (Atyame et al. 2011). Assuming a putative origin of both species from those regions, the introduction of mosquito populations possessing similar strains of *W. pipientis* (or no infection) may explain the high levels of hybridization in Cape Verde. Molecular analysis of *W. pipientis* in these mosquito populations would help clarifying this hypothesis.

Isolation between close species can be promoted by several mechanisms that may act in simultaneous. The lack or incomplete action of prezygotic (e.g. mating behavior) and intrinsic postzygotic (e.g. cytoplasmic incompatibility) mechanisms allows hybridization creating first generation (F1) hybrids. However, an extrinsic postzygotic mechanism such as hybrid sterility or hybrid low fitness can restrict gene flow to one generation avoiding introgression (Bono and Markow 2009; McBride and Singer 2010; Muñoz et al. 2010). The analysis performed by NEWHYBRIDS in Cape Verde samples showed the presence of ∼50% of second generation hybrids (25.6% F2 and 23.1% BxP; Table 3) within the hybrid cluster. The repeated hybridization and backcrossing with *Cx. pipiens* indicate mating success of F1 individuals (males and females) suggesting a low effect of extrinsic postzygotic isolation mechanisms between the *Cx. pipiens* and *Cx. quinquefasciatus* in Cape Verde.

Macaronesia is a passage route and breeding region for migratory birds (Garcia-del-Rey 2011). These birds may potentially introduce parasites and viruses that are known to be transmitted by the *Cx. pipiens* complex such as *Plasmodium relictum* (avian malaria), avian pox virus, Usutu virus and WNV. The introduction of these pathogens may place the endemic bird populations in danger (Kilpatrick 2011; Savini et al. 2011; Lapointe et al. 2012). Furthermore, the high levels of hybridization between *Cx. pipiens* and *Cx. quinquefasciatus* may promote a more opportunistic feeding behavior increasing the chance for the accidental transmission of WNV to humans. A serologic survey in 1980s showed 40% positive cases of WNV in Cape Verdean children (Vieira 1985). However, it has been recognized that WNV serologic surveys of the last century had considerable false positives due to cross-reactivity with other flavivirus (Tardei et al. 2000). Even so, the possibility of disease outbreaks should not be neglected given the outcome of the introduction of WNV to the western hemisphere (Kilpatrick 2011), or the more recent dengue epidemic in Cape Verde in 2009 (Franco et al. 2010), highlighting the receptivity of a territory once a suitable vector is present.

## Supporting Information

**Table S1.** Localities positive for *Culex pipiens* complex in Cape Verde.

**Table S2.** Microsatellite loci used in the analysis.

**Table S3.** Genetic diversity at microsatellite loci of *Culex pipiens* complex from Macaronesian islands.

**Figure S1.** Graphics of ad hoc approaches to infer the number of clusters (*K*) in STRUCTURE analysis with all samples, Cape Verde and Madeira.

**Figure S2.** Bayesian cluster analysis conducted by STRUCTURE in Madeira.

**Figure S3.** Graphics of ad hoc approaches to inference the number of clusters (*K*) in STRUCTURE analysis in each island of Cape Verde.
