# Supplementary material for: Hybridization and population structure of the Culex pipiens complex in the islands of Macaronesia
Source: Ecol Evol. 2012 Jul 6;2(8):1889–902. doi: 10.1002/ece3.307 (PMC3433992; doi:10.1002/ece3.307)
Supplement: Supplementary file 1 [file ece30002-1889-SD1.docx]

Table S1 – Localities positive for *Culex pipiens* complex in Cape Verde

| **Island** | **SS** | **locality** | **Type** | **PS** | **Coordinates** | **Altitude**  **(meters)** |
| --- | --- | --- | --- | --- | --- | --- |
| Brava | 7 | Travessa | Rural | 1 | 14º52’N 24º42’W | 504 |
|  |  | Figueira Grande | Rural | 1 | 14º52’N 24º42’W | 565 |
| Fogo | 12 | Patim | Rural | 2 | 14º52’N 24º25’W | 544 |
|  |  | Monte Largo | Rural | 1 | 14º52’N 24º22’W | 813 |
|  |  | Fonte Cabrito | Rural | 1 | 14º51’N 24º19’W | 657 |
| Maio | 18 | Morro | Rural | 1 | 15º11’N 23º13’W | 21 |
|  |  | Vila de Maio | Urban | 1 | 15º08’N 23º12’W | 48 |
| Santiago | 131 | Praia - Várzea | Urban | 5 | 14º54’N 23º30’W | 5 |
|  |  | Praia - Palmarejinho | Urban | 2 | 14º54’N 23º31’W | 5 |
|  |  | Praia – Eugénio Lima | Urban | 2 | 14º54’N 23º30’W | 5 |
|  |  | João Garrido | Rural | 2 | 15º01’N 23º34’W | 400* |
|  |  | Achada Leite | Rural | 1 | 15º07’N 23º45’W | 20* |
|  |  | Calhetona | Rural | 1 | 15º10’N 23º35’W | 44 |
|  |  | São Martinho Grande | Rural | 1 | 14º55’N 23º34’W | 50* |

SS: total number of breeding sites sampled per island; PS: positive sites for *Cx. pipiens* *sensu lato* per locality; * Estimated from Google^®^ Earth, remaining values were obtained from Global Positioning System measurements.

Table S2. Microsatellite loci used in the analysis

| Locus | Repeat | Primers | T_A_ (ºC) | Reference |
| --- | --- | --- | --- | --- |
| CQ11 | (GT)_2_ACTTC(GT)_9_ | F: GATCCTAGCAAGCGAGAAC  R: 6-fam-GAGCGGCCAAATATTGAGAC | 52 | Fonseca *et al.* 1998 |
| CQ26 | (GTGTGTAT)_2_+(GT)_10_+(GT)_5_ | F: TCCGACATGGGAAGAGCGCA  R: 6-fam-ACGCGCCCTTCTTCTGCAAC | 56 | Fonseca *et al.* 1998 |
| CQ41 | (GT)_12_ | F: CTGCCACTGCCTGACTGAAA  R: Hex-ACCACTCAGCAACATCCGGC | 52 | Fonseca *et al.* 1998 |
| CxpGT4 | (GT)_5_(GTTT)_2_GC(GT)_2_CT(GT)_5_ | F: GTCGTCGCTAACCCTTGTT  R: Ned-CGCGATAGTCGGTAATCGT | 54 | Keyghobadi *et al.* 2004 |
| CxpGT9 | (GT)_13_ | F: AATCTCCCCGTATAATTGTG  R: Ned-TATAAGACCAGTGAAGCCAG | 52 | Keyghobadi *et al.* 2004 |
| CxpGT12 | (TG)_14_ | F: AACGTGAGCGTGATTGCTC  R: 6-fam-CAGCTGTTGCACCAATGTC | 54 | Keyghobadi *et al.* 2004 |
| CxpGT40 | (GT)_15_ | F: CATCATCTGTCCACGATCC  R: Hex-TTATGCAGTTGCTGTCATATCC | 52 | Keyghobadi *et al.* 2004 |
| CxpGT46 | (TG)15 | F: Hex-CCGACACCGTGTTCAAAGAG  R: TGACGACGACGGTACAAGAG | 52 | Keyghobadi *et al.* 2004 |
| CxpGT51 | (TG)_4_CG(TG)_15_ | F: GAGTATCGCTCGTTGGAGATT  R: Hex-ACCCTCTTTTCTTTCTATGTCTGT | 54 | Keyghobadi *et al.* 2004 |
| CxqGT4 | (GT)_12_ | F: ATAGAACTTGTTCGCCGTCTC  R: 6-fam-TCTAAACACGCACCACGTACA | 52 | Smith *et al.* 2005 |
| CxqGT6b | (CA)_8_ | F: CAACCAGCAAAACCCTCATC  R: Ned-TAGCCGGGCAGATTCATTAC | 54 | Smith *et al.* 2005 |
| CxqTri4 | (TGC)_7_ | F: Hex-CTAGCCCGGTATTTACAAGAAC  R: AACGCCAGTAGTCTCAGCAG | 54 | Smith *et al.* 2005 |

T_A_: annealing temperature. Fonseca *et al.* 1998. *Molecular Ecology* 7: 1613-1621. Keyghobadi *et al.* 2004. *Molecular Ecology Notes* 4: 20-22. Smith *et al.* 2005. *Molecular Ecology Notes* 5: 697–700.

Table S3 - Genetic diversity at microsatellite loci of *Culex pipiens* complex from Macaronesian Islands

| Locus | Local | SC  (N=34) | RB  (N=39) | SA  (N=51) | PM  (N=66) | MAD  (N=190) | B  (N=31) | F  (N=36) | | M  (N=63) | | | S  (N=54) | CV  (N=184) | Total  (N=374) |
| --- | --- | --- | --- | --- | --- | --- | --- | --- | --- | --- | --- | --- | --- | --- | --- |
|  | ACE-2 assay | P  (N=34) | P  (N=39) | P  (N=51) | P  (N=66) |  | Q  (N=31) | Q  (N=22) | H  (N=14) | Q  (N=8) | H  (N=42) | P  (N=13) | Q  (N=54) |  |  |
| CQ11 | *A_R(16)_* | 3.1 | 2.2 | 3.2 | 2.9 | 3.0 | 1.5 | 3.7 | 3.5 | 3.0 | 3.7 | 3.6 | 2.9 | 3.8 | 4.9 |
|  | *H_e_* | 0.536 | **0.310*** | **0.581*** | **0.518*** | **0.509** | 0.063 | 0.429 | 0.452 | 0.700 | 0.594 | 0.525 | 0.458 | **0.600** | **0.763** |
|  | *F_IS_* | 0.337 | 0.681 | **0.563** | **0.433** | **0.492** | -0.017 | 0.424 | 0.500 | -0.077 | -0.166 | 0.376 | -0.133 | **0.288** | **0.546** |
| CQ26 | *A_R(16)_* | 4.2 | 4.5 | 4.5 | 3.0 | 4.0 | 3.0 | 3.9 | 3.6 | 4.0 | 5.6 | 4.8 | 3.4 | 5.9 | 7.4 |
|  | *H_e_* | **0.604*** | **0.723*** | **0.622*** | **0.523*** | **0.610** | 0.552 | 0.648 | 0.640 | 0.767 | **0.773** | 0.803 | 0.615 | **0.791** | **0.850** |
|  | *F_IS_* | **0.693** | **0.518** | **0.456** | **0.708** | **0.591** | -0.232 | 0.018 | 0.226 | -0.333 | 0.076 | -0.157 | -0.175 | 0.100 | **0.435** |
| CQ41 | *A_R(16)_* | 7.0 | 7.6 | 7.4 | 7.0 | 7.6 | 3.8 | 3.4 | 4.1 | 4.0 | 3.2 | 4.0 | 4.1 | 4.3 | 8.0 |
|  | *H_e_* | **0.846*** | **0.874*** | **0.864*** | **0.844*** | **0.874** | 0.635 | **0.614** | 0.593 | 0.708 | 0.542 | 0.462 | 0.706 | **0.733** | **0.869** |
|  | *F_IS_* | **0.482** | **0.416** | **0.516** | **0.261** | **0.413** | 0.036 | 0.339 | 0.161 | 0.125 | -0.277 | -0.175 | 0.162 | **0.199** | **0.367** |
| CxpGT04 | *A_R(16)_* | 3.5 | 4.3 | 3.4 | 4.1 | 3.9 | 1.3 | 3.2 | 4.0 | 3.0 | 3.8 | 3.8 | 1.1 | 3.7 | 5.4 |
|  | *H_e_* | 0.496 | 0.611 | 0.609 | 0.704 | 0.636 | 0.032 | 0.595 | 0.481 | 0.342 | **0.688** | 0.588 | 0.019 | **0.474** | **0.764** |
|  | *F_IS_* | -0.008 | -0.134 | 0.022 | -0.020 | -0.013 | 0.000 | 0.085 | -0.195 | -0.105 | 0.171 | -0.049 | 0.000 | **0.347** | **0.378** |
| CxpGT09 | *A_R(16)_* | 4.5 | 4.7 | 5.0 | 4.6 | 4.8 | 1.0 | 1.9 | 1.8 | 3.0 | 2.7 | 2.0 | 1.8 | 3.3 | 6.0 |
|  | *H_e_* | 0.736 | 0.730 | 0.773 | 0.752* | 0.756 | NA | 0.224 | 0.138 | 0.633 | **0.456*** | 0.271 | **0.123*** | **0.580** | **0.827** |
|  | *F_IS_* | 0.041 | 0.052 | 0.087 | 0.175 | **0.109** | NA | 0.782 | 1,000 | 0.622 | **0.689** | -0.143 | **0.851** | **0.858** | **0.522** |
| CxpGT12 | *A_R(16)_* | 3.3 | 3.5 | 3.6 | 2.8 | 3.3 | 1.0 | 2.2 | 2.0 | 2.0 | 3.0 | 3.0 | 1.1 | 2.8 | 3.6 |
|  | *H_e_* | 0.590 | 0.565 | 0.639 | 0.553 | 0.584 | NA | 0.210 | 0.423 | 0.525 | 0.669 | 0.655 | 0.019 | **0.412** | **0.669** |
|  | *F_IS_* | 0.103 | 0.139 | 0.235 | 0.207 | **0.180** | NA | 0.357 | -0.013 | -0.207 | -0.178 | 0.304 | 0.000 | **0.281** | **0.418** |
| CxpGT40 | *A_R(16)_* | 6.0 | 4.9 | 5.6 | 4.6 | 5.4 | 2.0 | 3.0 | 2.6 | 4.0 | 6.4 | 7.1 | 2.4 | 5.3 | 6.5 |
|  | *H_e_* | 0.785 | 0.738 | 0.785 | 0.668 | 0.745 | **0.373*** | 0.357 | 0.548 | 0.742 | **0.847** | **0.886** | 0.530 | **0.698** | **0.803** |
|  | *F_IS_* | 0.036 | 0.063 | -0.014 | 0.025 | 0.039 | **0.744** | 0.112 | -0.600 | -0.012 | 0.016 | -0.043 | 0.147 | **0.226** | **0.216** |
| CxpGT46 | *A_R(16)_* | 4.5 | 4.1 | 4.0 | 3.9 | 4.3 | 2.6 | 2.9 | 2.9 | 3.0 | 4.6 | 4.0 | 3.0 | 4.3 | 5.3 |
|  | *H_e_* | 0.673 | 0.667 | **0.627*** | 0.586 | **0.633** | 0.392 | 0.532 | 0.558 | 0.433 | **0.700** | **0.692** | 0.494 | **0.570** | **0.761** |
|  | *F_IS_* | 0.131 | 0.273 | 0.338 | -0.035 | **0.167** | 0.012 | 0.235 | 0.498 | -0.167 | -0.021 | 0.342 | 0.238 | **0.186** | **0.349** |
| CxpGT51 | *A_R(16)_* | 7.1 | 8.0 | 6.7 | 7.5 | 7.4 | 4.1 | 2.9 | 3.0 | 4.0 | 6.4 | 7.0 | 4.8 | 7.0 | 8.3 |
|  | *H_e_* | 0.817* | 0.838 | 0.830 | 0.856 | 0.843 | 0.685 | 0.517 | 0.627 | 0.725 | **0.830** | **0.846** | 0.747 | **0.844** | **0.873** |
|  | *F_IS_* | 0.224 | -0.010 | 0.007 | -0.062 | 0.022 | -0.229 | -0.057 | -0.509 | -0.225 | -0.208 | 0.373 | 0.009 | **0.053** | **0.070** |
| CxqQGT4 | *A_R(16)_* | 1.2 | 1.4 | 1.4 | 1.0 | 1.2 | 3.3 | 2.3 | 2.0 | 2.0 | 3.0 | 3.0 | 2.8 | 3.8 | 3.6 |
|  | *H_e_* | 0.029 | 0.051 | 0.058 | NA | 0.031 | 0.441 | 0.292 | 0.389 | 0.400 | **0.638** | 0.566 | 0.423 | **0.534** | **0.601** |
|  | *F_IS_* | 0.000 | -0.013 | -0.020 | NA | -0.013 | 0.017 | -0.148 | 0.085 | -0.273 | 0.181 | -0.379 | 0.109 | **0.162** | **0.610** |
| CxqGT6B | *A_R(16)_* | 2.9 | 2.6 | 2.6 | 2.8 | 2.7 | 4.4 | 3.6 | 4.6 | 4.0 | 3.0 | 1.6 | 2.9 | 4.4 | 4.1 |
|  | *H_e_* | 0.510 | 0.505 | 0.384 | 0.496 | 0.472 | 0.757 | 0.511 | 0.730 | 0.617 | **0.586** | 0.077 | 0.408 | **0.729** | **0.643** |
|  | *F_IS_* | -0.166 | -0.095 | 0.031 | -0.193 | -0.112 | -0.110 | -0.162 | -0.079 | -0.235 | -0.388 | 0.000 | -0.229 | **0.121** | **0.095** |
| CxqTRI4 | *A_R(16)_* | 2.0 | 2.0 | 2.2 | 2.0 | 2.0 | 1.8 | 3.0 | 2.8 | 2.0 | 2.0 | 2.0 | 1.9 | 2.4 | 3.2 |
|  | *H_e_* | 0.506 | 0.506 | 0.441 | 0.490 | 0.488 | 0.178 | 0.623 | 0.561 | 0.325 | 0.496 | 0.369 | 0.124 | **0.467** | **0.675** |
|  | *F_IS_* | -0.019 | 0.089 | 0.156 | -0.083 | 0.036 | -0.091 | 0.127 | 0.112 | 0.632 | 0.137 | -0.263 | -0.044 | **0.337** | **0.421** |
| All loci | *A_R(16)_* | 4.1 | 4.2 | 4.1 | 3.9 | 4.1 | 2.5 | 3.0 | 3.1 | 3.2 | 4.0 | 3.8 | 2.7 | 4.3 | 5.5 |
|  | *H_e_* | **0.594** | **0.593** | **0.601** | **0.636** | **0.599** | 0.411 | 0.463 | 0.512 | 0.576 | 0.652 | 0.562 | 0.389 | **0.619** | **0.758** |
|  | *F_IS_* | **0.183** | **0.169** | **0.219** | **0.122** | **0.177** | -0.017 | 0.144 | 0.033 | -0.032 | -0.011 | 0.051 | 0.043 | **0.244** | **0.365** |

*A_R(16)_*: allelic richness for a minimum sample size of 16 genes; *H_e_*: expected heterozygosity; *F_IS_*: inbreeding coefficient; *N*: sample size; SC: Santa Cruz; RB: Ribeira Brava; SA: Santana; PM: Porto Moniz; MAD: Madeira island; B: Brava island; F: Fogo island; M: Maio island; S: Santiago island; CV: Cape Verde; P: *Cx. pipiens*; Q: *Cx. quinquefasciatus;* H: hybrids (ACE-2 based identification). In bold: significant p-values for H-W tests (heterozygote deficit) after correction for multiple tests. Asterisks indicate presence of null alleles determined by Micro-Checker. Per locus and over samples H-W tests were performed in ARLEQUIN. For over loci estimates the global test available in FSTAT was used.


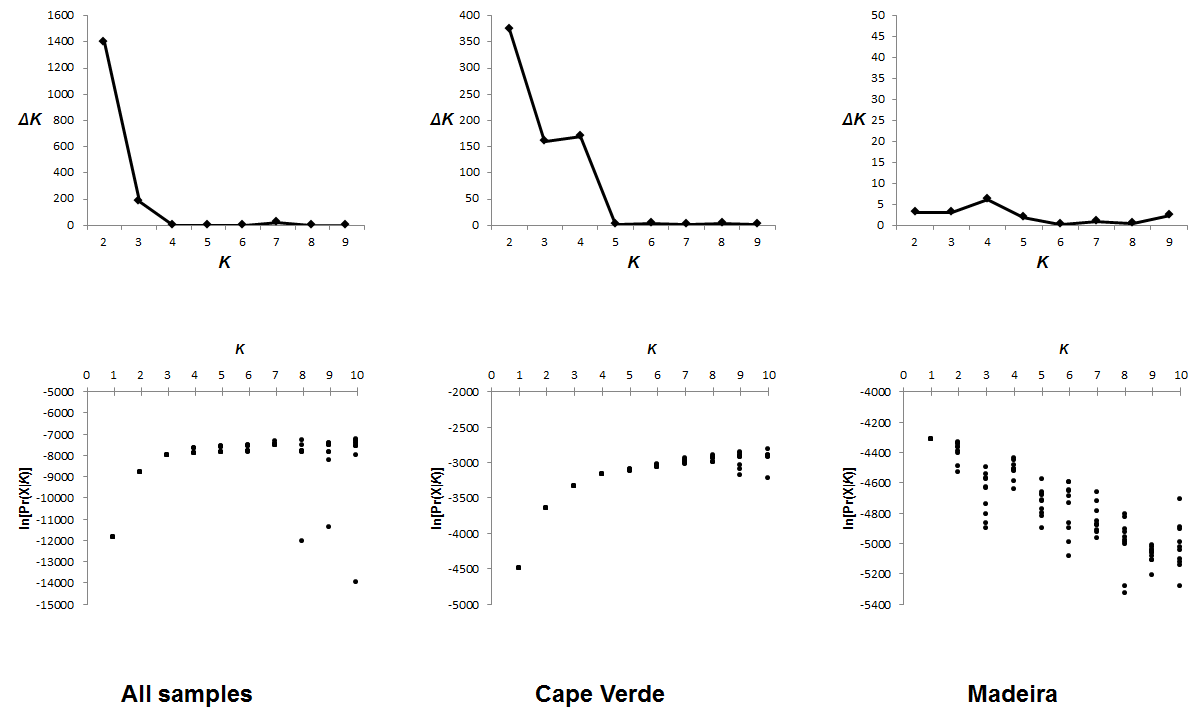


Figure S1 – Graphics of *ad hoc* approaches to infer the number of clusters (*K*) in STRUCTURE analysis with all samples, Cape Verde and Madeira.

*K*: number of clusters; Δ*K*: see Evanno *et al.* (2005); ln[Pr(X|*K*)]: estimated log probability of the data under each *K.*


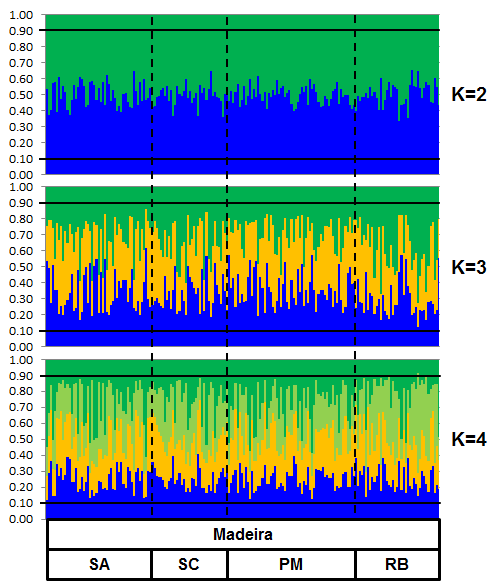


Figure S2 - Bayesian cluster analysis conducted by STRUCTURE in Madeira

*K*: number of clusters; SA: Santana; SC: Santa Cruz; PM: Porto Moniz; RB: Ribeira Brava. Columns correspond to the multilocus genotype of each individual, partitioned in different colours representing the probability of ancestry (*q_i_*) to each cluster. Individuals were ordered according to their geographic information. Horizontal lines indicate the *q_i_* threshold used to determine admixed individuals (see Methods).


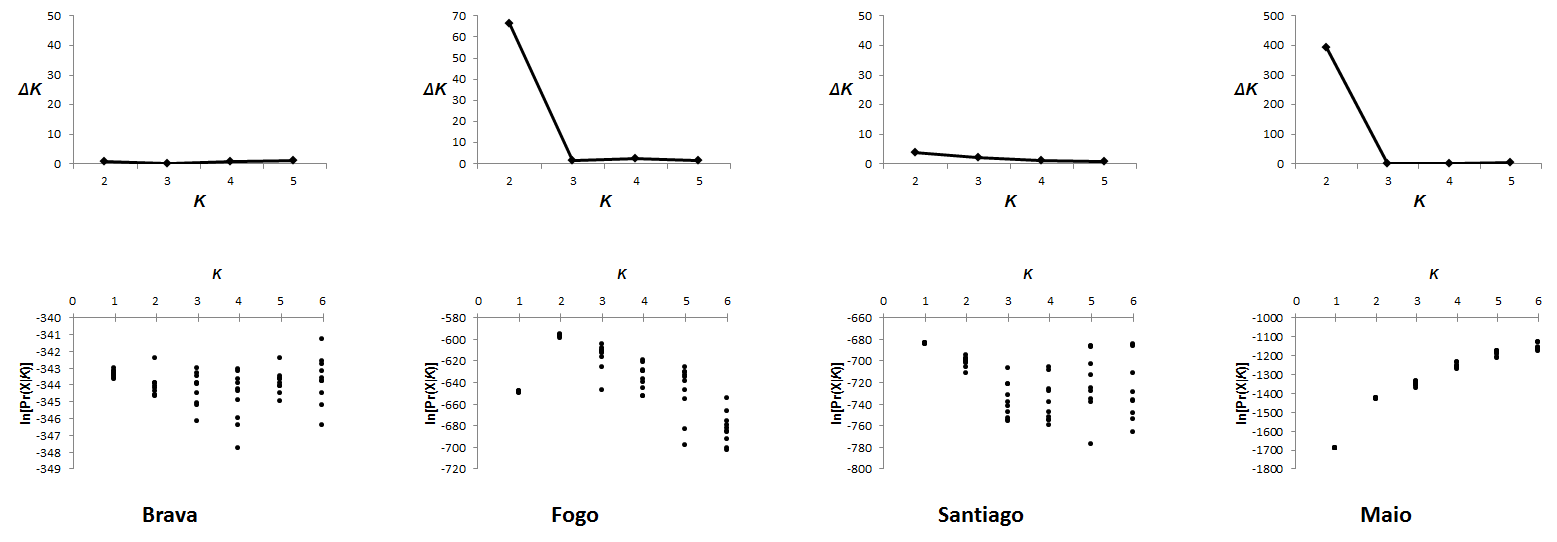


Figure S3 - Graphics of *ad hoc* approaches to inference the number of clusters (*K*) in STRUCTURE analysis in each island of Cape Verde.

*K*: number of clusters; Δ*K*: see Evanno *et al.* 2005. *Molecular Ecology* 14: 2611-2620; ln[Pr(X|*K*)]: estimated log probability of the data under each *K.*
